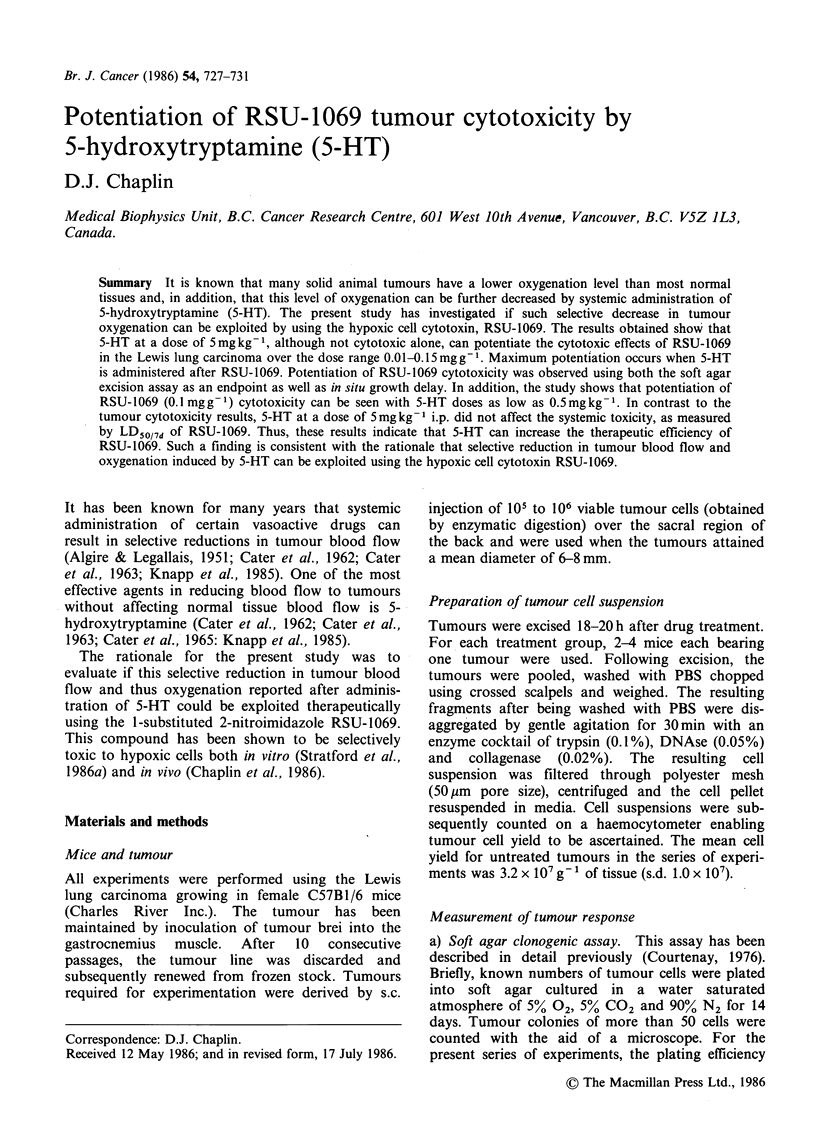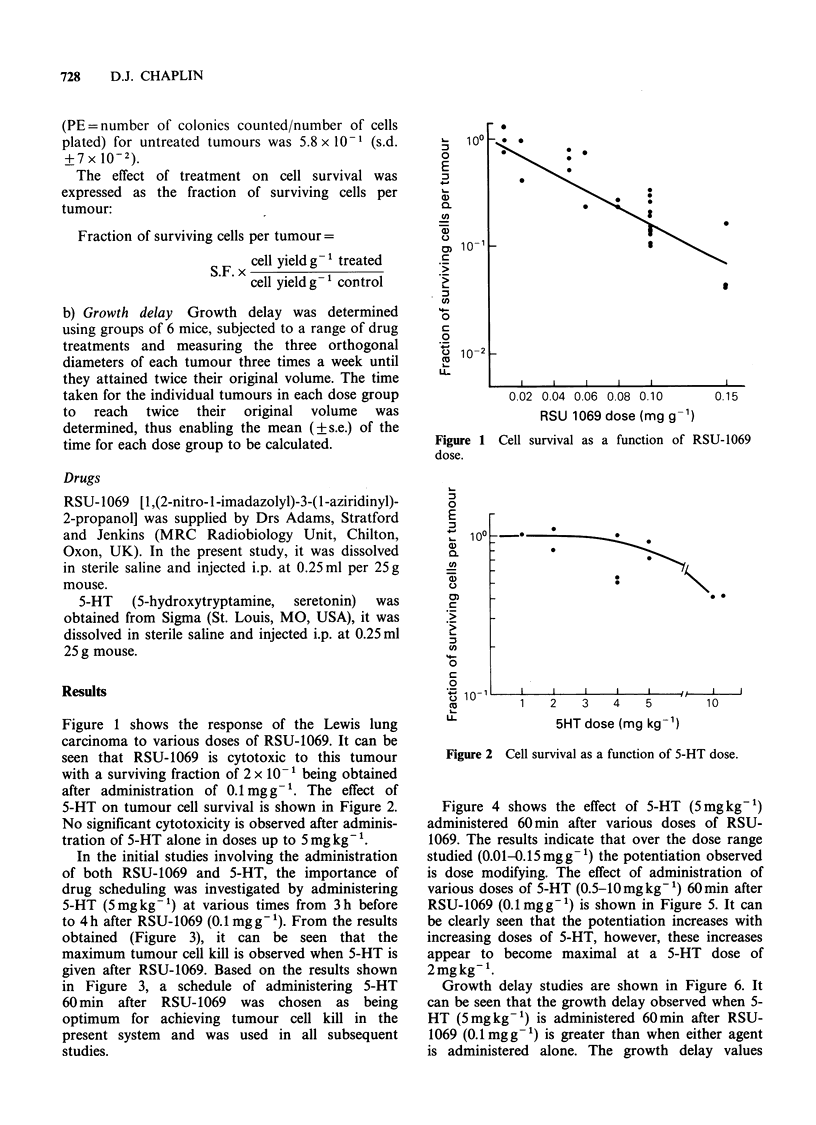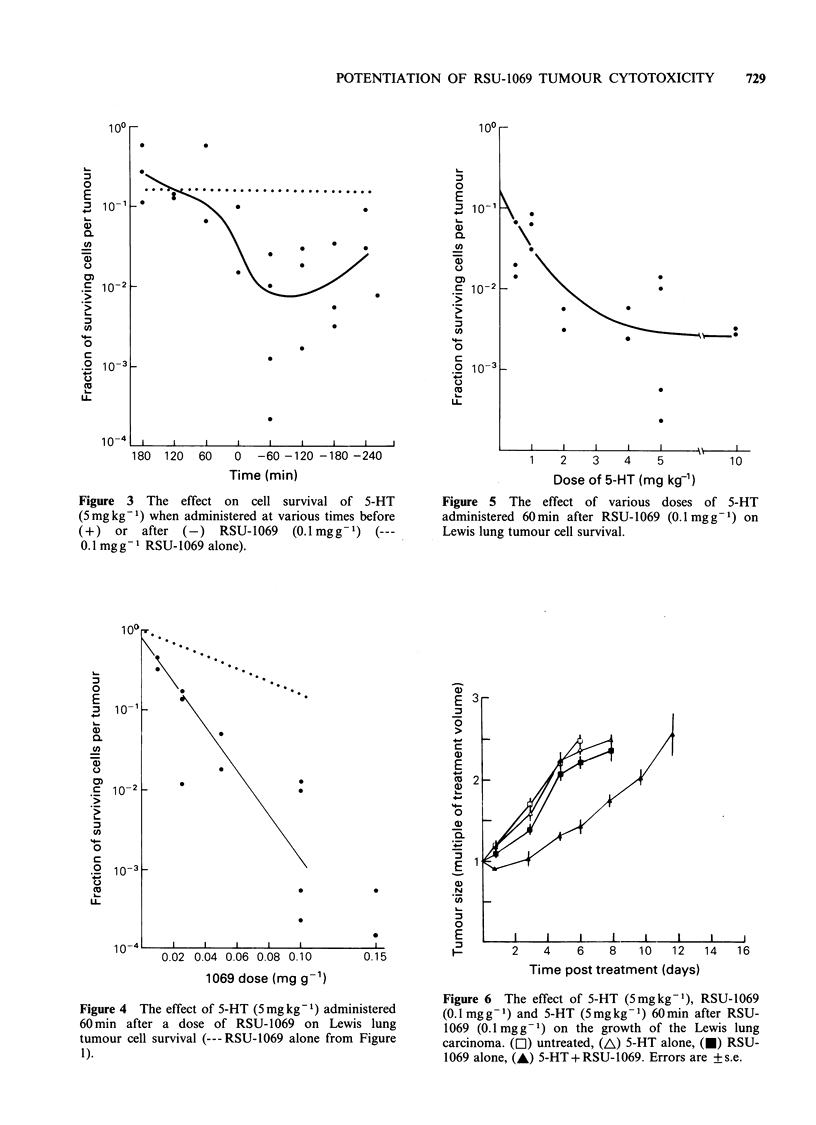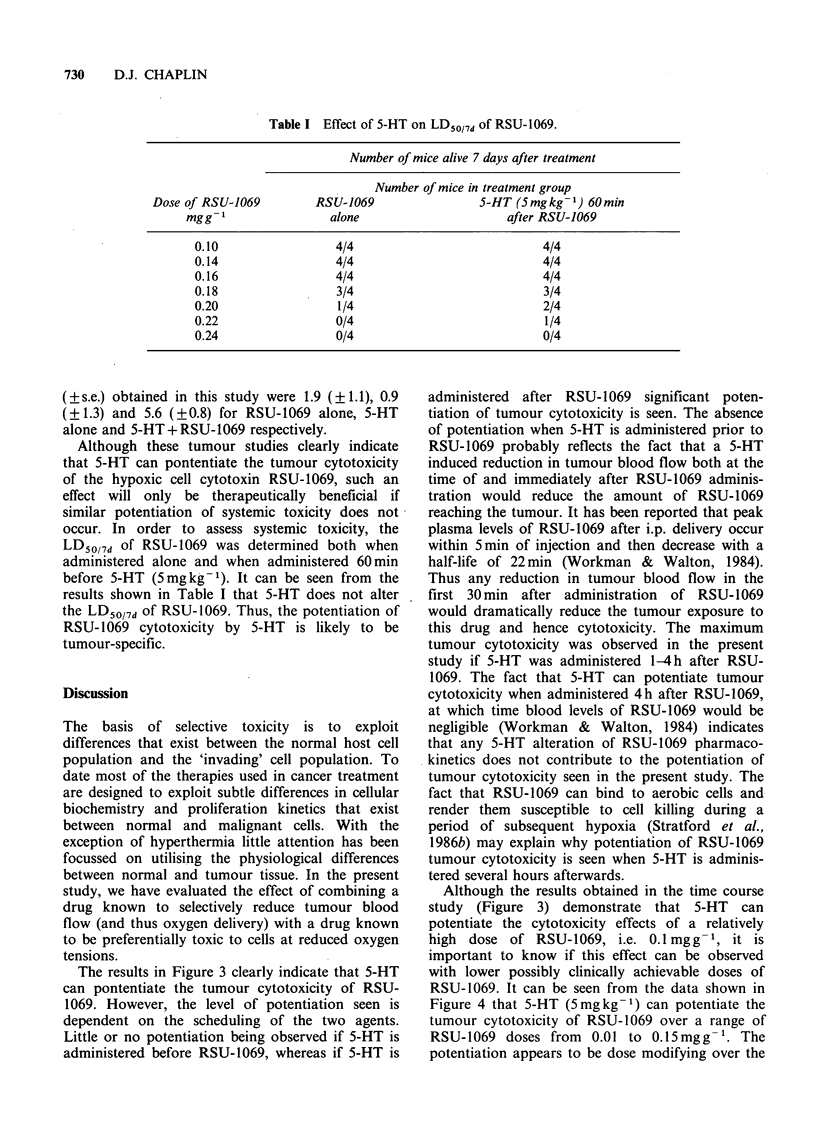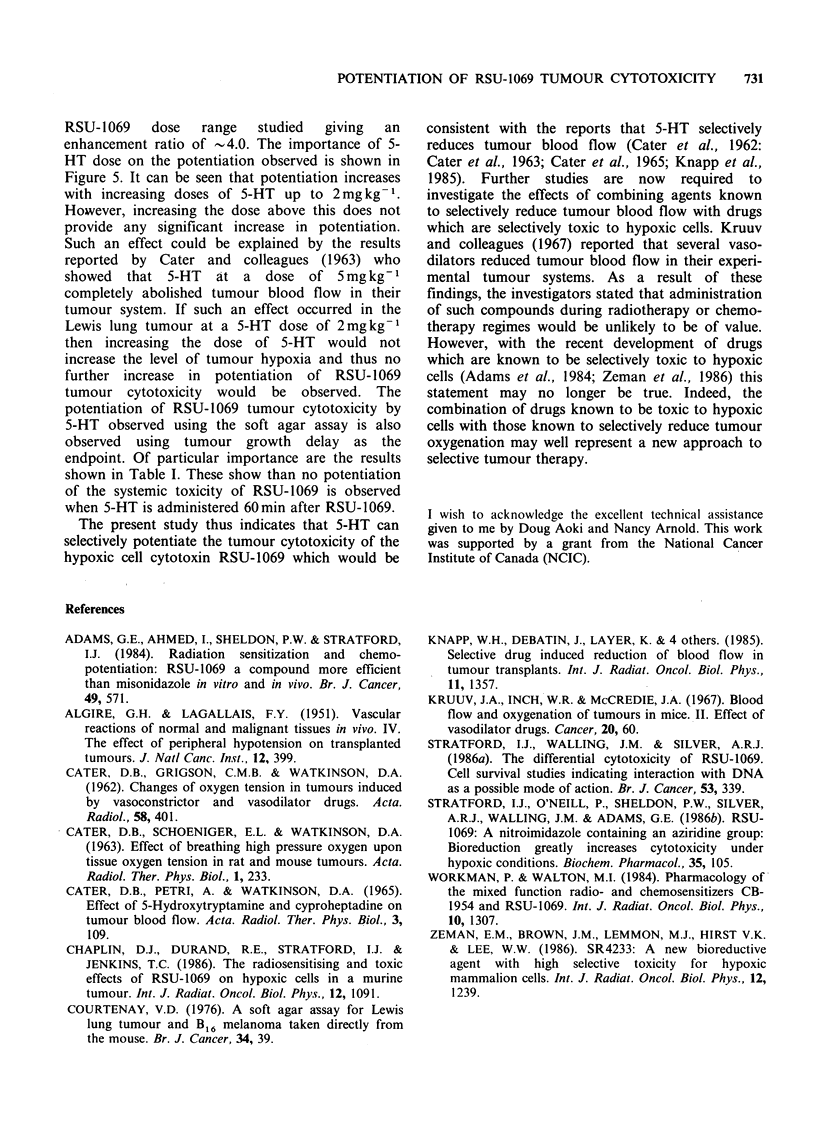# Potentiation of RSU-1069 tumour cytotoxicity by 5-hydroxytryptamine (5-HT).

**DOI:** 10.1038/bjc.1986.233

**Published:** 1986-11

**Authors:** D. J. Chaplin

## Abstract

It is known that many solid animal tumours have a lower oxygenation level than most normal tissues and, in addition, that this level of oxygenation can be further decreased by systemic administration of 5-hydroxytryptamine (5-HT). The present study has investigated if such selective decrease in tumour oxygenation can be exploited by using the hypoxic cell cytotoxin, RSU-1069. The results obtained show that 5-HT at a dose of 5 mg kg-1, although not cytotoxic alone, can potentiate the cytotoxic effects of RSU-1069 in the Lewis lung carcinoma over the dose range 0.01-0.15 mg g-1. Maximum potentiation occurs when 5-HT is administered after RSU-1069. Potentiation of RSU-1069 cytotoxicity was observed using both the soft agar excision assay as an endpoint as well as in situ growth delay. In addition, the study shows that potentiation of RSU-1069 (0.1 mg g-1) cytotoxicity can be seen with 5-HT doses as low as 0.5 mg kg-1. In contrast to the tumour cytotoxicity results, 5-HT at a dose of 5 mg kg-1 i.p. did not affect the systemic toxicity, as measured by LD50/7d of RSU-1069. Thus, these results indicate that 5-HT can increase the therapeutic efficiency of RSU-1069. Such a finding is consistent with the rationale that selective reduction in tumour blood flow and oxygenation induced by 5-HT can be exploited using the hypoxic cell cytotoxin RSU-1069.


Br. J. Cancer (1986) 54, 727–731

### Potentiation of RSU-1069 tumour cytotoxicity by 5-hydroxytryptamine (5-HT)

D.J. Chaplin

*Medical Biophysics Unit, B.C. Cancer Research Centre, 601 West 10th Avenue, Vancouver, B.C. V5Z 1L3, Canada.*

**Summary** It is known that many solid animal tumours have a lower oxygenation level than most normal tissues and, in addition, that this level of oxygenation can be further decreased by systemic administration of 5-hydroxytryptamine (5-HT). The present study has investigated if such selective decrease in tumour oxygenation can be exploited by using the hypoxic cell cytotoxin, RSU-1069. The results obtained show that 5-HT at a dose of $5\,\mathrm{mg\,kg^{-1}}$, although not cytotoxic alone, can potentiate the cytotoxic effects of RSU-1069 in the Lewis lung carcinoma over the dose range $0.01$–$0.15\,\mathrm{mg\,g^{-1}}$. Maximum potentiation occurs when 5-HT is administered after RSU-1069. Potentiation of RSU-1069 cytotoxicity was observed using both the soft agar excision assay as an endpoint as well as *in situ* growth delay. In addition, the study shows that potentiation of RSU-1069 ($0.1\,\mathrm{mg\,g^{-1}}$) cytotoxicity can be seen with 5-HT doses as low as $0.5\,\mathrm{mg\,kg^{-1}}$. In contrast to the tumour cytotoxicity results, 5-HT at a dose of $5\,\mathrm{mg\,kg^{-1}}$ i.p. did not affect the systemic toxicity, as measured by $LD_{50/7d}$ of RSU-1069. Thus, these results indicate that 5-HT can increase the therapeutic efficiency of RSU-1069. Such a finding is consistent with the rationale that selective reduction in tumour blood flow and oxygenation induced by 5-HT can be exploited using the hypoxic cell cytotoxin RSU-1069.

It has been known for many years that systemic administration of certain vasoactive drugs can result in selective reductions in tumour blood flow (Algire & Legallais, 1951; Cater *et al.*, 1962; Cater *et al.*, 1963; Knapp *et al.*, 1985). One of the most effective agents in reducing blood flow to tumours without affecting normal tissue blood flow is 5-hydroxytryptamine (Cater *et al.*, 1962; Cater *et al.*, 1963; Cater *et al.*, 1965: Knapp *et al.*, 1985).

The rationale for the present study was to evaluate if this selective reduction in tumour blood flow and thus oxygenation reported after administration of 5-HT could be exploited therapeutically using the 1-substituted 2-nitroimidazole RSU-1069. This compound has been shown to be selectively toxic to hypoxic cells both *in vitro* (Stratford *et al.*, 1986*a*) and *in vivo* (Chaplin *et al.*, 1986).

### Materials and methods

### *Mice and tumour*

All experiments were performed using the Lewis lung carcinoma growing in female C57B1/6 mice (Charles River Inc.). The tumour has been maintained by inoculation of tumour brei into the gastrocnemius muscle. After 10 consecutive passages, the tumour line was discarded and subsequently renewed from frozen stock. Tumours required for experimentation were derived by s.c. injection of $10^5$ to $10^6$ viable tumour cells (obtained by enzymatic digestion) over the sacral region of the back and were used when the tumours attained a mean diameter of 6–8 mm.

### *Preparation of tumour cell suspension*

Tumours were excised 18–20 h after drug treatment. For each treatment group, 2–4 mice each bearing one tumour were used. Following excision, the tumours were pooled, washed with PBS chopped using crossed scalpels and weighed. The resulting fragments after being washed with PBS were disaggregated by gentle agitation for 30 min with an enzyme cocktail of trypsin (0.1%), DNAse (0.05%) and collagenase (0.02%). The resulting cell suspension was filtered through polyester mesh (50 $\mu$m pore size), centrifuged and the cell pellet resuspended in media. Cell suspensions were subsequently counted on a haemocytometer enabling tumour cell yield to be ascertained. The mean cell yield for untreated tumours in the series of experiments was $3.2\times10^7\,\mathrm{g^{-1}}$ of tissue (s.d. $1.0\times10^7$).

### *Measurement of tumour response*

a) *Soft agar clonogenic assay.* This assay has been described in detail previously (Courtenay, 1976). Briefly, known numbers of tumour cells were plated into soft agar cultured in a water saturated atmosphere of 5% $O_2$, 5% $CO_2$ and 90% $N_2$ for 14 days. Tumour colonies of more than 50 cells were counted with the aid of a microscope. For the present series of experiments, the plating efficiency

Correspondence: D.J. Chaplin.
Received 12 May 1986; and in revised form, 17 July 1986.

© The Macmillan Press Ltd., 1986

(PE = number of colonies counted/number of cells plated) for untreated tumours was $5.8 \times 10^{-1}$ (s.d. $\pm 7 \times 10^{-2}$).

The effect of treatment on cell survival was expressed as the fraction of surviving cells per tumour:

$$\text{Fraction of surviving cells per tumour} = \text{S.F.} \times \frac{\text{cell yield g}^{-1}\text{ treated}}{\text{cell yield g}^{-1}\text{ control}}$$

b) *Growth delay* Growth delay was determined using groups of 6 mice, subjected to a range of drug treatments and measuring the three orthogonal diameters of each tumour three times a week until they attained twice their original volume. The time taken for the individual tumours in each dose group to reach twice their original volume was determined, thus enabling the mean ($\pm$s.e.) of the time for each dose group to be calculated.

*Drugs*

RSU-1069 [1,(2-nitro-1-imadazolyl)-3-(1-aziridinyl)-2-propanol] was supplied by Drs Adams, Stratford and Jenkins (MRC Radiobiology Unit, Chilton, Oxon, UK). In the present study, it was dissolved in sterile saline and injected i.p. at 0.25 ml per 25 g mouse.

5-HT (5-hydroxytryptamine, seretonin) was obtained from Sigma (St. Louis, MO, USA), it was dissolved in sterile saline and injected i.p. at 0.25 ml 25 g mouse.

### Results

Figure 1 shows the response of the Lewis lung carcinoma to various doses of RSU-1069. It can be seen that RSU-1069 is cytotoxic to this tumour with a surviving fraction of $2 \times 10^{-1}$ being obtained after administration of $0.1\,\text{mg g}^{-1}$. The effect of 5-HT on tumour cell survival is shown in Figure 2. No significant cytotoxicity is observed after administration of 5-HT alone in doses up to $5\,\text{mg kg}^{-1}$.

In the initial studies involving the administration of both RSU-1069 and 5-HT, the importance of drug scheduling was investigated by administering 5-HT ($5\,\text{mg kg}^{-1}$) at various times from 3 h before to 4 h after RSU-1069 ($0.1\,\text{mg g}^{-1}$). From the results obtained (Figure 3), it can be seen that the maximum tumour cell kill is observed when 5-HT is given after RSU-1069. Based on the results shown in Figure 3, a schedule of administering 5-HT 60 min after RSU-1069 was chosen as being optimum for achieving tumour cell kill in the present system and was used in all subsequent studies.

**Figure 1** Cell survival as a function of RSU-1069 dose.

**Figure 2** Cell survival as a function of 5-HT dose.

Figure 4 shows the effect of 5-HT ($5\,\text{mg kg}^{-1}$) administered 60 min after various doses of RSU-1069. The results indicate that over the dose range studied ($0.01$–$0.15\,\text{mg g}^{-1}$) the potentiation observed is dose modifying. The effect of administration of various doses of 5-HT ($0.5$–$10\,\text{mg kg}^{-1}$) 60 min after RSU-1069 ($0.1\,\text{mg g}^{-1}$) is shown in Figure 5. It can be clearly seen that the potentiation increases with increasing doses of 5-HT, however, these increases appear to become maximal at a 5-HT dose of $2\,\text{mg kg}^{-1}$.

Growth delay studies are shown in Figure 6. It can be seen that the growth delay observed when 5-HT ($5\,\text{mg kg}^{-1}$) is administered 60 min after RSU-1069 ($0.1\,\text{mg g}^{-1}$) is greater than when either agent is administered alone. The growth delay values

**Figure 3** The effect on cell survival of 5-HT ($5\,mg\,kg^{-1}$) when administered at various times before (+) or after (−) RSU-1069 ($0.1\,mg\,g^{-1}$) (--- $0.1\,mg\,g^{-1}$ RSU-1069 alone).

**Figure 5** The effect of various doses of 5-HT administered 60 min after RSU-1069 ($0.1\,mg\,g^{-1}$) on Lewis lung tumour cell survival.

**Figure 4** The effect of 5-HT ($5\,mg\,kg^{-1}$) administered 60 min after a dose of RSU-1069 on Lewis lung tumour cell survival (--- RSU-1069 alone from Figure 1).

**Figure 6** The effect of 5-HT ($5\,mg\,kg^{-1}$), RSU-1069 ($0.1\,mg\,g^{-1}$) and 5-HT ($5\,mg\,kg^{-1}$) 60 min after RSU-1069 ($0.1\,mg\,g^{-1}$) on the growth of the Lewis lung carcinoma. (□) untreated, (△) 5-HT alone, (■) RSU-1069 alone, (▲) 5-HT + RSU-1069. Errors are ±s.e.

**Table I** Effect of 5-HT on $LD_{50/7d}$ of RSU-1069.

| | *Number of mice alive 7 days after treatment* | |
|---|---|---|
| | *Number of mice in treatment group* | |
| *Dose of RSU-1069* $mg\,g^{-1}$ | *RSU-1069 alone* | *5-HT* ($5\,mg\,kg^{-1}$) *60 min after RSU-1069* |
| 0.10 | 4/4 | 4/4 |
| 0.14 | 4/4 | 4/4 |
| 0.16 | 4/4 | 4/4 |
| 0.18 | 3/4 | 3/4 |
| 0.20 | 1/4 | 2/4 |
| 0.22 | 0/4 | 1/4 |
| 0.24 | 0/4 | 0/4 |

($\pm$s.e.) obtained in this study were 1.9 ($\pm$1.1), 0.9 ($\pm$1.3) and 5.6 ($\pm$0.8) for RSU-1069 alone, 5-HT alone and 5-HT + RSU-1069 respectively.

Although these tumour studies clearly indicate that 5-HT can pontentiate the tumour cytotoxicity of the hypoxic cell cytotoxin RSU-1069, such an effect will only be therapeutically beneficial if similar potentiation of systemic toxicity does not occur. In order to assess systemic toxicity, the $LD_{50/7d}$ of RSU-1069 was determined both when administered alone and when administered 60 min before 5-HT ($5\,mg\,kg^{-1}$). It can be seen from the results shown in Table I that 5-HT does not alter the $LD_{50/7d}$ of RSU-1069. Thus, the potentiation of RSU-1069 cytotoxicity by 5-HT is likely to be tumour-specific.

### Discussion

The basis of selective toxicity is to exploit differences that exist between the normal host cell population and the 'invading' cell population. To date most of the therapies used in cancer treatment are designed to exploit subtle differences in cellular biochemistry and proliferation kinetics that exist between normal and malignant cells. With the exception of hyperthermia little attention has been focussed on utilising the physiological differences between normal and tumour tissue. In the present study, we have evaluated the effect of combining a drug known to selectively reduce tumour blood flow (and thus oxygen delivery) with a drug known to be preferentially toxic to cells at reduced oxygen tensions.

The results in Figure 3 clearly indicate that 5-HT can pontentiate the tumour cytotoxicity of RSU-1069. However, the level of potentiation seen is dependent on the scheduling of the two agents. Little or no potentiation being observed if 5-HT is administered before RSU-1069, whereas if 5-HT is administered after RSU-1069 significant potentiation of tumour cytotoxicity is seen. The absence of potentiation when 5-HT is administered prior to RSU-1069 probably reflects the fact that a 5-HT induced reduction in tumour blood flow both at the time of and immediately after RSU-1069 administration would reduce the amount of RSU-1069 reaching the tumour. It has been reported that peak plasma levels of RSU-1069 after i.p. delivery occur within 5 min of injection and then decrease with a half-life of 22 min (Workman & Walton, 1984). Thus any reduction in tumour blood flow in the first 30 min after administration of RSU-1069 would dramatically reduce the tumour exposure to this drug and hence cytotoxicity. The maximum tumour cytotoxicity was observed in the present study if 5-HT was administered 1–4 h after RSU-1069. The fact that 5-HT can potentiate tumour cytotoxicity when administered 4 h after RSU-1069, at which time blood levels of RSU-1069 would be negligible (Workman & Walton, 1984) indicates that any 5-HT alteration of RSU-1069 pharmacokinetics does not contribute to the potentiation of tumour cytotoxicity seen in the present study. The fact that RSU-1069 can bind to aerobic cells and render them susceptible to cell killing during a period of subsequent hypoxia (Stratford *et al.*, 1986*b*) may explain why potentiation of RSU-1069 tumour cytotoxicity is seen when 5-HT is administered several hours afterwards.

Although the results obtained in the time course study (Figure 3) demonstrate that 5-HT can potentiate the cytotoxicity effects of a relatively high dose of RSU-1069, i.e. $0.1\,mg\,g^{-1}$, it is important to know if this effect can be observed with lower possibly clinically achievable doses of RSU-1069. It can be seen from the data shown in Figure 4 that 5-HT ($5\,mg\,kg^{-1}$) can potentiate the tumour cytotoxicity of RSU-1069 over a range of RSU-1069 doses from 0.01 to $0.15\,mg\,g^{-1}$. The potentiation appears to be dose modifying over the

RSU-1069 dose range studied giving an enhancement ratio of $\sim$4.0. The importance of 5-HT dose on the potentiation observed is shown in Figure 5. It can be seen that potentiation increases with increasing doses of 5-HT up to $2 mg kg^{-1}$. However, increasing the dose above this does not provide any significant increase in potentiation. Such an effect could be explained by the results reported by Cater and colleagues (1963) who showed that 5-HT at a dose of $5 mg kg^{-1}$ completely abolished tumour blood flow in their tumour system. If such an effect occurred in the Lewis lung tumour at a 5-HT dose of $2 mg kg^{-1}$ then increasing the dose of 5-HT would not increase the level of tumour hypoxia and thus no further increase in potentiation of RSU-1069 tumour cytotoxicity would be observed. The potentiation of RSU-1069 tumour cytotoxicity by 5-HT observed using the soft agar assay is also observed using tumour growth delay as the endpoint. Of particular importance are the results shown in Table I. These show than no potentiation of the systemic toxicity of RSU-1069 is observed when 5-HT is administered 60 min after RSU-1069.

The present study thus indicates that 5-HT can selectively potentiate the tumour cytotoxicity of the hypoxic cell cytotoxin RSU-1069 which would be consistent with the reports that 5-HT selectively reduces tumour blood flow (Cater *et al.*, 1962: Cater *et al.*, 1963; Cater *et al.*, 1965; Knapp *et al.*, 1985). Further studies are now required to investigate the effects of combining agents known to selectively reduce tumour blood flow with drugs which are selectively toxic to hypoxic cells. Kruuv and colleagues (1967) reported that several vasodilators reduced tumour blood flow in their experimental tumour systems. As a result of these findings, the investigators stated that administration of such compounds during radiotherapy or chemotherapy regimes would be unlikely to be of value. However, with the recent development of drugs which are known to be selectively toxic to hypoxic cells (Adams *et al.*, 1984; Zeman *et al.*, 1986) this statement may no longer be true. Indeed, the combination of drugs known to be toxic to hypoxic cells with those known to selectively reduce tumour oxygenation may well represent a new approach to selective tumour therapy.

I wish to acknowledge the excellent technical assistance given to me by Doug Aoki and Nancy Arnold. This work was supported by a grant from the National Cancer Institute of Canada (NCIC).

### References

ADAMS, G.E., AHMED, I., SHELDON, P.W. & STRATFORD, I.J. (1984). Radiation sensitization and chemopotentiation: RSU-1069 a compound more efficient than misonidazole *in vitro* and *in vivo*. *Br. J. Cancer*, **49**, 571.

ALGIRE, G.H. & LAGALLAIS, F.Y. (1951). Vascular reactions of normal and malignant tissues *in vivo*. IV. The effect of peripheral hypotension on transplanted tumours. *J. Natl Canc. Inst.*, **12**, 399.

CATER, D.B., GRIGSON, C.M.B. & WATKINSON, D.A. (1962). Changes of oxygen tension in tumours induced by vasoconstrictor and vasodilator drugs. *Acta. Radiol.*, **58**, 401.

CATER, D.B., SCHOENIGER, E.L. & WATKINSON, D.A. (1963). Effect of breathing high pressure oxygen upon tissue oxygen tension in rat and mouse tumours. *Acta. Radiol. Ther. Phys. Biol.*, **1**, 233.

CATER, D.B., PETRI, A. & WATKINSON, D.A. (1965). Effect of 5-Hydroxytryptamine and cyproheptadine on tumour blood flow. *Acta. Radiol. Ther. Phys. Biol.*, **3**, 109.

CHAPLIN, D.J., DURAND, R.E., STRATFORD, I.J. & JENKINS, T.C. (1986). The radiosensitising and toxic effects of RSU-1069 on hypoxic cells in a murine tumour. *Int. J. Radiat. Oncol. Biol. Phys.*, **12**, 1091.

COURTENAY, V.D. (1976). A soft agar assay for Lewis lung tumour and $B_{16}$ melanoma taken directly from the mouse. *Br. J. Cancer*, **34**, 39.

KNAPP, W.H., DEBATIN, J., LAYER, K. & 4 others. (1985). Selective drug induced reduction of blood flow in tumour transplants. *Int. J. Radiat. Oncol. Biol. Phys.*, **11**, 1357.

KRUUV, J.A., INCH, W.R. & McCREDIE, J.A. (1967). Blood flow and oxygenation of tumours in mice. II. Effect of vasodilator drugs. *Cancer*, **20**, 60.

STRATFORD, I.J., WALLING, J.M. & SILVER, A.R.J. (1986*a*). The differential cytotoxicity of RSU-1069. Cell survival studies indicating interaction with DNA as a possible mode of action. *Br. J. Cancer*, **53**, 339.

STRATFORD, I.J., O'NEILL, P., SHELDON, P.W., SILVER, A.R.J., WALLING, J.M. & ADAMS, G.E. (1986*b*). RSU-1069: A nitroimidazole containing an aziridine group: Bioreduction greatly increases cytotoxicity under hypoxic conditions. *Biochem. Pharmacol.*, **35**, 105.

WORKMAN, P. & WALTON, M.I. (1984). Pharmacology of the mixed function radio- and chemosensitizers CB-1954 and RSU-1069. *Int. J. Radiat. Oncol. Biol. Phys.*, **10**, 1307.

ZEMAN, E.M., BROWN, J.M., LEMMON, M.J., HIRST V.K. & LEE, W.W. (1986). SR4233: A new bioreductive agent with high selective toxicity for hypoxic mammalion cells. *Int. J. Radiat. Oncol. Biol. Phys.*, **12**, 1239.